# Patient–doctor continuity and diagnosis of cancer: electronic medical records study in general practice

**DOI:** 10.3399/bjgp15X684829

**Published:** 2015-04-27

**Authors:** Matthew J Ridd, Diana L Santos Ferreira, Alan A Montgomery, Chris Salisbury, William Hamilton

**Affiliations:** Centre for Academic Primary Care, School of Social and Community Medicine, University of Bristol, Bristol.; Centre for Academic Primary Care, School of Social and Community Medicine, University of Bristol, Bristol.; Nottingham Clinical Trials Unit, University of Nottingham, Nottingham.; Centre for Academic Primary Care, School of Social and Community Medicine, University of Bristol, Bristol.; University of Exeter, Exeter.

**Keywords:** cancer, continuity of care, diagnosis, general practice, patient-doctor continuity, symptoms

## Abstract

**Background:**

Continuity of care may affect the diagnostic process in cancer but there is little research.

**Aim:**

To estimate associations between patient–doctor continuity and time to diagnosis and referral of three common cancers.

**Design and setting:**

Retrospective cohort study in general practices in England.

**Method:**

This study used data from the General Practice Research Database for patients aged ≥40 years with a diagnosis of breast, colorectal, or lung cancer. Relevant cancer symptoms or signs were identified up to 12 months before diagnosis. Patient–doctor continuity (fraction-of-care index adjusted for number of consultations) was calculated up to 24 months before diagnosis. Time ratios (TRs) were estimated using accelerated failure time regression models.

**Results:**

Patient–doctor continuity in the 24 months before diagnosis was associated with a slightly later diagnosis of colorectal (time ratio [TR] 1.01, 95% confidence interval [CI] =1.01 to 1.02) but not breast (TR = 1.00, 0.99 to 1.01) or lung cancer (TR = 1.00, 0.99 to 1.00). Secondary analyses suggested that for colorectal and lung cancer, continuity of doctor before the index consultation was associated with a later diagnosis but continuity after the index consultation was associated with an earlier diagnosis, with no such effects for breast cancer. For all three cancers, most of the delay to diagnosis occurred after referral.

**Conclusion:**

Any effect for patient–doctor continuity appears to be small. Future studies should compare investigations, referrals, and diagnoses in patients with and without cancer who present with possible cancer symptoms or signs; and focus on ‘difficult to diagnose’ types of cancer.

## INTRODUCTION

Continuity of care is a core value in general practice. It is a multifaceted concept, but one key aspect valued by patients is continuity of doctor; being able to see the same GP over time for multiple problems.^1^ In the UK, a combination of changes in the way primary care services are organised and doctors work means that patient–doctor continuity has been eroded.^2^ Seeing the same doctor is associated with higher patient satisfaction but evidence that it makes a difference to patient outcomes is weak.^3^,^4^

The value of being able to see the same doctor appears to depend on the nature of the problem.^5^ From the doctor’s perspective, familiarity with the patient may be especially helpful when dealing with new or ‘unspecified’ symptoms, such as fatigue.^6^ This may be particularly relevant to the diagnosis of cancers in primary care, where in the initial stages of the disease symptoms may be very non-specific and/or may be explained by coexisting physical or psychological conditions. Conversely, it is possible that familiarity with the patient and their problems may mean doctors misattribute new complaints to ongoing problems or personality traits, leading to delayed or missed diagnoses.^5^,^7^

Surprisingly, there is little research published on the influence of patient–doctor continuity on the diagnostic process in primary care, and even less in the field of cancer diagnostics.^4^ Therefore, this study investigated the relationship between patient–doctor continuity in general practice and time to diagnosis of lung, colorectal, and breast cancer in adults.

## METHOD

### Study data

Data were used from the General Practice Research Database, now known as the Clinical Practice Research Datalink, from 1 January 2000 to 31 December 2009 inclusive. General practices contribute to the database from around the UK and adhere to stringent data quality recording standards.^8^

Patients had an incident breast, colorectal, or lung cancer; were ≥40 years at diagnosis; and had ≥1 year of prior registration data. These cancers were chosen because they are common and GP personal knowledge of the patient may be more (for example, fatigue in colorectal or lung cancer) or less useful (for example, breast cancer, most commonly presenting with a breast lump). Patients were excluded if they had an in-situ cancer, their recorded date of death was before or the same as their diagnosis date, or they were asymptomatic (no recorded cancer symptom/sign) in the 12 months before diagnosis. Analyses were further restricted to consultations where symptoms or signs were recorded in consultations with GPs (partner, salaried, registrar, or locum) in relevant types of encounter (mainly surgery, telephone, or home visit). The few male patients in the breast cancer dataset were also excluded.

**How this fits in**

Continuity of care is a core value in general practice yet, nowadays, patients are less likely to see the same doctor. It is unknown whether seeing the same doctor leads to a faster or slower diagnosis of cancer among patients who present with symptoms. Overall, this study found that any effect of patient–doctor continuity on time to diagnosis of breast, colorectal, or lung cancer was small. While GPs should be cautious not to dismiss potentially significant symptoms or signs among patients they know well, it may be prudent for doctors to personally follow-up patients with ‘low-risk but not no-risk’ symptoms.

Relevant symptoms and signs (classified as high-risk or low-risk) for each cancer (Table 1) were based on the National Institute for Health and Care Excellence *Referral Guidelines for Suspected Cancer*.^9^ These were updated by reference to recent systematic reviews on colorectal cancer^10^ and breast cancer;^11^ and a case–control study of lung cancer.^12^ Symptoms and signs were identified using Read Codes only, which were independently identified and agreed by the GPs on the team. High-risk took precedence over low-risk symptoms or signs where both were recorded in the index consultation. The presence or absence of risk factors for each type of cancer were also identified: family history (breast cancer); ulcerative colitis (colorectal cancer); and current/ex-smoker or chronic obstructive pulmonary disease (lung cancer). Referrals, appropriate to each cancer, were identified for ‘definitive’ investigations (for example colonoscopy for colorectal cancer) or secondary care opinion (for example respiratory physician for lung cancer).

**Table 1.** High-risk and low-risk cancer symptoms/signs for breast, colorectal, and lung cancer

**Risk type**	**Cancer symptoms or signs**		
	**Breast**	**Colorectal**	**Lung**
**High-risk**	Breast lump Nipple eczema Nipple distortion Nipple discharge (bloody)	Rectal bleeding Change in bowel habit, looser stools and/or increased stool frequency Abdominal or rectal mass	Haemoptysis Stridor Superior vena cava obstruction

**Low-risk**	Breast skin changes	Weight loss	Chest pain
	Breast pain	Fatigue	Shoulder pain
		Abdominal pain	Dyspnoea
		Anorexia	Hoarseness
		Constipation	Cough
		Diarrhoea	Chest signs
			Finger clubbing
			Cervical or supraclavicular lymphadenopathy
			Fatigue
			Weight loss

The list of diagnostic codes used has been developed previously as part of DISCOVERY (http://discovery-programme.org/; a 5-year programme of work designed to improve the diagnosis of cancer) and has supported several publications.^13^–^18^ Patient multimorbidity was quantified by a simple count of 17 chronic diseases included in the clinical domain of the Quality and Outcomes Framework, as at 2007–2008, using methods described previously.^19^

### Patient–doctor continuity

The index consultation (and hence the index doctor) was defined as the first consultation in the 12 months before diagnosis when a relevant cancer symptom or sign was recorded by a GP. Patients needed at least one other contact with the index GP in the 24 months before diagnosis to be included in the study.

Patient–doctor continuity was summarised using the fraction-of-care (*f*) index, which is the proportion of doctor encounters during a continuity defining period that were made to the current provider (that is, the index GP). Because *f* is sensitive to utilisation levels (that is, people who visit infrequently), it was adjusted for the number of consultations in all analyses (*f′*). In the statistical models, *f′* was multiplied by 10 so that the regression coefficients represent the change in outcome associated with a 10% difference in continuity.

In the primary analysis the effect of patient–doctor continuity was explored during the whole 24 months before diagnosis. In secondary analyses, the intervals were examined separately before the index consultation and after the index consultation.

### Outcomes

This study investigated the effect of patient–doctor continuity on time to diagnosis and time to referral, expressed as the number of days from the first recorded sign or symptom of cancer until date of cancer diagnosis or date of referral.

Time to diagnosis (or diagnostic interval) was chosen as the primary end-point because the date of diagnosis is usually easily determined; previous studies have shown an effect of organisational change (that is, introduction of ‘2-week wait’ system) on this interval;^20^ and it allows the findings to be easily compared with most other studies.^21^

Time to referral (to relevant secondary care specialty or for definitive investigation) was explored as a secondary end-point because events after the referral (which are within the control of secondary care rather than primary care) may cause delays between referral and diagnosis. Where referral was made on the same day as the index consultation (around one-third of patients), 1 day was added so that the statistical model could be fitted.

### Analysis

All analyses were carried out using Stata (version 12). First, a simple descriptive analysis was undertaken to examine the characteristics of participating patients, doctors, and their practices; patient–doctor consultation rates; number and type of symptoms/signs at the index consultation; and patient–doctor continuity before and after the index consultation.

Next, regression models (accelerated-failure time) were constructed to examine univariable and multivariable associations between patient–doctor continuity and time to diagnosis and time to referral. The accelerated failure time model is a parametric model that provides an alternative to proportional hazards models commonly used in time-to-event analyses.^22^ It allows the derivation of a time ratio, which is more readily interpretable than a ratio of two hazards generated by other survival analysis approaches: a time ratio >1 for the covariate implies that it prolongs the time to the event, while a time ratio <1 indicates that an earlier event is more likely.^23^ Plots were constructed to check model assumptions. Log-normal, log-logistic, generalised gamma, and Weibull distributions were used to represent the survival data. The Akaike information criterion measure of the goodness of fit of an estimated statistical model was used to select the best model.

Alternative models for time to referral and time to diagnosis, using the different continuity defining periods, were constructed. The following covariates were included in each model: patient age, sex, multimorbidity, and cancer-specific risk factor(s); index doctor sex and status; index consultation type; and number of symptoms/signs at index consultation. Interactions between patient–doctor continuity and symptom/sign type (high/low risk) were added to the models, but none with a likelihood ratio test <0.05 were found. The extent of clustering by practice was estimated and adjusted for as necessary in all models.

## RESULTS

### Characteristics of participants and patients’ consultations

Table 2 shows the initial and final number of patients (with a relevant cancer, symptoms/signs in the 12 months prior to diagnosis, and qualifying consultations) analysed in each cancer dataset. The characteristics of participants and patients’ consultations are given in Table 3. With respect to cancer risk factors, 81 (2.7%) of patients with breast cancer had a family history of breast cancer; 91 (1.2%) of patients with colorectal cancer had ulcerative colitis; and 1845 (22.7%) of patients with lung cancer had a history of chronic obstructive pulmonary disease, with about one-third being current smokers (*n* = 2639, 32.4%) and one-half ex-smokers (*n* = 4079, 50.1%).

**Table 2.** Construction of cancer datasets for analysis

	**Patients in each cancer dataset, *n* (%)**		
	**Breast**	**Colorectal**	**Lung**
**Initial dataset (combined clinical and referral data)**	11 251 (100.0)	17 390 (100.0)	17 097 (100.0)

**Ineligible diagnoses**			
In-situ cancers	1080 (9.6)	1554 (8.9)	1213 (7.1)
Date of death recorded before diagnosis or on same day	35 (0.3)	51 (0.3)	108 (0.6)
Asymptomatic patients	7066 (62.8)	8071 (46.4)	7229 (42.3)

**Ineligible consultations**			
No previous encounter with index GP	51 (0.5)	272 (1.6)	354 (2.1)
Index GP sex unknown	50 (0.4)	48 (0.3)	50 (0.3)
Patient male^a^	14 (0.1)	n/a	n/a

**Patients included in analysis**	**2955 (26.3)**	**7394 (42.5)**	**8143 (47.6)**

a Breast cancer analysis was restricted to female patients only. n/a = not applicable.

**Table 3.** Characteristics of participants and patients’ consultations

**Characteristic**	**Patients in each cancer dataset, *n* (%)**		
	**Breast**	**Colorectal**	**Lung**
**Practices, *n***	465	475	476

**Doctors, *n***	1836	2564	2843
GP partners, *n* (%)	1635 (89.1)	2181 (85.1)	2412 (84.8)
Female, n (%)	971 (52.9)	1214 (47.3)	1425 (50.1)

**Patients, *n***	2955	7394	8143
Mean age, years (SD)	66.7 (14.1)	71.4 (11.3)	71.5 (10.2)
Female, *n* (%)	2955 (100.0)	3442 (46.6)	3471 (42.6)
Mean multimorbidity score (SD)	1.2 (1.3)	1.3 (1.3)	1.3 (1.3)

**Patient–doctor consultations^a^**			
Mean number (SD)	15.3 (13.7)	16.6 (13.1)	19.9 (14.3)
Continuity (*f′*)	0.58 (0.23)	0.62 (0.21)	0.66 (0.18)

**Cancer symptoms/signs**			
Total	2989	7648	8531
Mean (SD) per patient	1.0 (0.1)	1.0 (0.2)	1.1 (0.2)
High risk			
- No. (%) by symptom/sign total	2805 (93.8)	1507 (19.7)	647 (7.6)
- No. (%) by consultations	2797 (94.2)	2528 (34.2)	636 (7.8)

Median time to referral in days (IQR)	0 (0–0)	8 (0–51)	36 (7–117)

Median time to diagnosis in days (IQR)	23 (14–42)	80 (36–174)	104 (44–223)

a During 24 months before diagnosis. IQR = interquartile range. f = fraction-of-care index.

Patients with breast cancer were more likely to present initially with at least one high-risk symptom or sign (*n* = 2797, 94.2%) than those with subsequent colorectal (*n* = 2528, 34.2%) or lung (*n* = 636, 7.8%) cancer diagnoses (Table 3); and (*n* = 2559, 86.6%) of patients with breast cancer were referred on the same day as the index consultation.

### Patient–doctor continuity and diagnosis of breast, colorectal, and lung cancer

The crude and adjusted associations with time to diagnosis for patient–doctor continuity, symptoms/signs and patient, doctor, and consultation characteristics for the three different cancers are shown in Table 4. There was no evidence of any association between patient–doctor continuity and time to diagnosis for breast cancer (adjusted TR_breast_ = 1.00, 95% CI = 0.99 to 1.01, *P* = 0.90) or lung cancer (adjusted TR_lung_ = 1.00, 95% CI = 0.99 to 1.00, *P* = 0.33). The adjusted TR of 1.01 (95% CI = 1.01 to 1.02, *P*<0.01) for colorectal cancer suggests there was a 1% increase in the diagnostic interval for every 10% increase in continuity. The factor most consistently associated with time to diagnosis across the different cancers was a high-risk symptom/sign being recorded at the index consultation (adjusted TR_breast_ = 0.34, 95% CI = 0.29 to 0.39, *P*<0.01; TR_colorectal_ = 0.72, 95% CI = 0.69 to 0.75, *P*<0.01; TR_lung_ = 0.70, 95% CI = 0.65 to 0.74, *P*<0.01).

**Table 4.** Crude and adjusted associations between patient–doctor continuity and time to diagnosis of breast, colorectal, and lung cancer

**Covariate**	**Breast**				**Colorectal**				**Lung**			
		
	**Crude time ratio (95% CI)**	***P*-value**	**Adjusted time ratio (95% CI)**	***P*-value**	**Crude time ratio (95% CI)**	***P*-value**	**Adjusted time ratio (95% CI)**	***P*-value**	**Crude time ratio (95% CI)**	***P*-value**	**Adjusted time ratio (95% CI)**	***P*-value**
Patient–doctor continuity^a^	1.01 (1.00 to 1.02)	0.28	1.00 (0.99 to 1.01)	0.90	1.00 (0.99 to 1.00)	0.21	1.01 (1.01 to 1.02)	<0.01	0.98 (0.98 to 0.99)	<0.01	1.00 (0.99 to 1.00)	0.33

Number of consultations	1.00 (1.00 to 1.00)	0.67	1.00 (1.00 to 1.00)	0.07	1.02 (1.02 to 1.02)	<0.01	1.02 (1.01 to 1.02)	<0.01	1.01 (1.01 to 1.02)	<0.01	1.01 (1.01 to 1.01)	<0.01

**Patient**												
Age	0.99 (0.99 to 0.99)	<0.01	0.99 (0.99 to 1.00)	<0.01	1.00 (1.00 to 1.00)	<0.01	1.00 (1.00 to 1.00)	<0.01	1.00 (1.00 to 1.00)	<0.01	1.00 (1.00 to 1.00)	0.20

Female	n/a		n/a		1.08 (1.04 to 1.13)	<0.01	1.05 (1.01 to 1.09)	0.03	1.06 (1.02 to 1.10)	<0.01	1.03 (0.99 to 1.07)	0.12

Multimorbidity	0.95 (0.93 to 0.97)	<0.01	0.97 (0.95 to 1.00	0.04	1.06 (1.04 to 1.08)	<0.01	0.99 (0.98 to 1.01)	0.48	1.06 (1.05 to 1.08)	<0.01	1.02 (1.01 to 1.04)	<0.01

Risk factor^b^	0.96 (0.79 to 1.15)	0.64	0.92 (0.77 to 1.10)	0.35	1.22 (1.02 to 1.47)	0.03	1.15 (0.96 to 1.37)	0.13	1.30 (1.24 to 1.35)	<0.01	1.19 (1.14 to 1.24)	<0.01

Current smoker	n/a		n/a		n/a		n/a		0.95 (0.92 to 0.99)	0.02	0.90 (0.85 to 0.95)	<0.01

Ex-smoker	n/a		n/a		n/a		n/a		1.03 (0.99 to 1.07)	0.13	0.91 (0.86 to 0.95)	<0.01

**Index doctor**												
Female	0.92 (0.87 to 0.98)	0.01	0.92 (0.86 to 0.98)	0.01	1.02 (0.98 to 1.07)	0.25	1.03 (0.99 to 1.08)	0.15	1.02 (0.99 to 1.07)	0.17	1.00 (0.96 to 1.04)	0.96

**Status**												
Salaried	0.92 (0.68 to 1.25)	0.59	0.91 (0.67 to 1.23)	0.46	0.94 (0.74 to 1.19)	0.72	0.86 (0.68 to 1.09)	0.45	0.99 (0.80 to 1.24)	0.08	1.02 (0.82 to 1.27)	0.15
Locum	1.05 (0.93 to 1.20)		1.07 (0.94 to 1.21)		0.97 (0.89 to 1.06)		1.00 (0.92 to 1.10)		0.92 (0.85 to 0.99)		0.93 (0.86 to 1.00)	

**Consultation type**												
Surgery	1.14 (1.02 to 1.29)	0.04	1.13 (1.01 to 1.26)	0.16	1.00 (0.91 to 1.08)	0.17	0.99 (0.91 to 1.08)	0.19	1.07 (0.98 to 1.16)	<0.01	1.08 (0.99 to 1.17)	<0.01
Telephone	1.24 (0.94 to 1.63)		1.22 (0.94 to 1.58)		1.11 (0.98 to 1.27)		1.02 (0.90 to 1.16)		1.09 (0.97 to 1.24)		1.01 (0.90 to 1.14)	
Visit	0.99 (0.82 to 1.20)		1.15 (0.95 to 1.38)		0.98 (0.88 to 1.10)		0.91 (0.82 to 1.02)		0.96 (0.86 to 1.06)		0.92 (0.83 to 1.01)	

**Symptoms/signs**												
Number	1.38 (0.94 to 2.01)	0.10	1.42 (0.98 to 2.05)	0.06	0.89 (0.81 to 0.98)	0.03	0.96 (0.87 to 1.06)	0.44	0.99 (0.91 to 1.07)	0.74	0.99 (0.91 to 1.07)	0.75
High-risk	0.32 (0.28 to 0.37)	<0.01	0.34 (0.29 to 0.39)	<0.01	0.68 (0.65 to 0.71)	<0.01	0.72 (0.69 to 0.75)	<0.01	0.66 (0.61 to 0.70)	<0.01	0.70 (0.65 to 0.74)	<0.01

a Modified fraction-of-care index (f’); continuity defining period before and after index consultation.

b Breast cancer: family history of breast cancer; colorectal cancer: ulcerative colitis; lung cancer: chronic obstructive pulmonary disease. n/a = not applicable.

Further analysis examined whether there was a relationship between patient–doctor continuity before or after the index consultation and time to diagnosis (Table 5). There was no evidence of an effect for patient–doctor continuity on time to diagnosis over any continuity-defining period for breast cancer. There was some evidence that increased continuity *before* the index consultation increased the time to diagnosis for both colorectal cancer (adjusted TR_colorectal_ = 1.02, 95% CI = 1.01 to 1.02, *P*<0.01) and lung cancer (TR_lung_ = 1.01, 95% CI = 1.00 to 1.01, *P*<0.01). Conversely, there was evidence that seeing the same doctor *after* the index consultation reduced the delay to diagnosis (adjusted TR_colorectal_ = 0.98, 95% CI = 0.98 to 0.99, *P*<0.01; TR_lung_ = 0.98, 95% CI = 0.97 to 0.98, *P*<0.01).

**Table 5.** Adjusted associations between patient–doctor continuity for different continuity-defining periods and time to referral and time to diagnosis of breast, colorectal, and lung cancer

**Outcome**	**Continuity-defining period^a^**	**Time ratios (95% CI)**		

		**Breast cancer**	**Colorectal cancer**	**Lung cancer**
**Time to diagnosis**	Before and after (primary analysis)	1.00 (0.99 to 1.01) *P*= 0.90	1.01 (1.01 to 1.02) *P*<0.01	1.00 (0.99 to 1.00) *P*= 0.33
	Before	1.00 (0.99 to 1.01) *P*= 0.75	1.02 (1.01 to 1.02) *P*<0.01	1.01 (1.00 to 1.01) *P*<0.01
	After	0.99 (0.99 to 1.00) *P*= 0.26	0.98 (0.98 to 0.99) *P*<0.01	0.98 (0.97 to 0.98) *P*<0.01
**Time to referral**	Before	0.90 (0.85 to 0.95) *P*<0.01	0.99 (0.98 to 1.01) P = 0.44	1.01 (0.99 to 1.03) *P*= 0.34

a In relation to index consultation, up to 24 months pre-diagnosis.

Finally, evidence of an effect for patient–doctor continuity *before* the index consultation on time to referral was found for patients with breast cancer only (adjusted TR_breast_ = 0.90, 95% CI = 0.85 to 0.95, *P*<0.01) (Table 5).

## DISCUSSION

### Summary

Overall, patient–doctor continuity was not associated with clinically important changes in time to diagnosis for patients with breast, colorectal, or lung cancer. In the primary analyses, the association seen with later diagnosis of colorectal cancer equates to a maximum delay of around 7 days; while in the secondary analyses the maximum reduction in time to diagnosis for patients with colorectal or lung cancer who see the same doctor after the index consultation are up to 14 and 18 days, respectively. For all cancers, the most significant factor predicting earlier diagnosis was first presentation with a high-risk symptom or sign; and the greatest delay for diagnosis of all three cancers occurred after the patients had been referred.

### Strengths and limitations

This is the first study to explore, using a large, reliable, and validated dataset, the effect of patient–doctor continuity on the diagnostic process of three common cancers (breast, colorectal, and lung). However, the analyses were restricted to between 26.3%, (*n* = 2955, breast), and 47.6%, (*n* = 8143, lung) of the original datasets most patients were excluded because they had no relevant Read-Coded symptoms or signs in the 12 months before diagnosis. It is important to remember that the data for this study come from medical records whose primary purpose is clinical care, rather than research, so it is likely that relevant symptoms and signs in both included and excluded patients were not coded. In addition, the final route by which patients obtained their diagnoses is not known. A significant proportion of patients may have been diagnosed after being admitted through the emergency department, independent of their GP.^24^

This study highlights the methodological challenges of operationalising continuity in this type of research.^25^ It was decided to quantify continuity in relation to the doctor seen at the index consultation and, while other approaches are possible (for example, defining continuity in terms of ‘usual doctor’), the authors believe this is the most appropriate for the research question posed: ‘Does seeing the same GP (around time of first presentation of possible cancer symptoms or signs) reduce time to diagnosis of three common cancers?’. A modified form of an established continuity index (fraction-of-care) was used but the findings were the same when the analyses were repeated using another more widely used index (Continuity of Care; available from the authors on request).^26^

This study improves our understanding of the role of patient–doctor continuity in patients who present with symptoms and signs who are subsequently diagnosed with cancer but not those with other outcomes or diagnoses. Also, any association between patient–doctor continuity and earlier diagnosis will be affected by variation in individual doctors’ thresholds for investigating symptoms and making referrals. That is, if doctors who provide low continuity also have a high referral rate, their patients will have a short delay to diagnosis of cancer, but at the expense of a high number of referrals that do not lead to a cancer diagnosis.

### Comparison with existing literature

Several studies have examined the role of continuity in relation to cancer screening^27^–^30^ but the authors are aware of only two studies concerning diagnosis.^31^,^32^ Both were conducted in the US and neither found that continuity at a primary care level was associated with an earlier stage of cancer at diagnosis. The continuity literature provides reasons to support and explain the observation in this study that seeing a known doctor at first presentation appears to delay diagnosis, yet seeing the same doctor afterwards promotes earlier diagnosis. In the case of the former, familiarity with the patient and their problems may mean that doctors make assumptions and become closed to other diagnoses;^33^ the doctor may ‘fail to see the wood for the trees’ and misattribute symptoms or dismiss them.^5^ However, when seeing the same doctor afterwards, the doctor may assume greater responsibility for the patient in ensuring complaints are followed up and to ensure that symptoms are either explained or resolved.^5^ It is noteworthy that the mean number of consultations in the 12 months before diagnosis for each cancer is higher than might be expected for populations in these age groups,^34^ although there is a wide variation as reflected in the standard deviations. Consultation frequency itself may be a cause for concern in the pre-diagnosis period.^35^

### Implications for research and practice

Future studies should examine the value of patient–doctor continuity in relation to the investigations and referrals that doctors make for patients who present with possible cancer symptoms or signs who do and do not go on to be given a cancer diagnosis. Ideally, future research should be prospective and incorporate other important patient characteristics (disclosure of symptoms and signs) and doctor characteristics (tolerance of uncertainty and personal thresholds for organising investigations and referrals), so that the relationship between continuity and these other factors can be assessed comprehensively. Finally, it would be worth repeating this work in ‘hard to diagnose’ cancers, in particular, those which are associated with a larger number of consultations before referral.^36^,^37^

What should GPs and policy makers do meanwhile? In keeping with much of the continuity literature in relation to patient outcomes, this study does not provide strong evidence that patient–doctor continuity reduces the time to diagnosis of breast, colorectal, or lung cancer. Rather, it suggests that doctors working in primary care should be cautioned against overlooking potentially worrying symptoms or signs among patients who they know well. Previous work has highlighted the potential problems of ‘over-familiarity’ and the potential benefit of having a ‘fresh set of eyes’.^5^,^38^ However, that is not to negate the psychological benefits that some patients may derive from ‘following through’ a cancer diagnosis with the same GP. Until further work is carried out, it would seem sensible to recommend that practices encourage patients to follow new problems up with the same doctor, especially for patients whose symptoms or signs at the initial consultation may represent an underlying cancer but do not in themselves warrant immediate investigation or referral.

Finally, although much attention has been given to reducing delays to referral from general practice for patients with symptoms suggestive of cancer, these data suggest that more attention should be given to the process of care between referral and diagnosis. This is the main source of delay and where there is most scope for reductions in the time to diagnosis.
